# Impact of Different Developmental Instars on *Locusta migratoria* Jumping Performance

**DOI:** 10.1155/2020/2797486

**Published:** 2020-03-19

**Authors:** Xiaojuan Mo, Donato Romano, Mario Milazzo, Giovanni Benelli, Wenjie Ge, Cesare Stefanini

**Affiliations:** ^1^School of Mechanical Engineering, Northwestern Polytechnical University, 710072 Xi'an, China; ^2^The BioRobotics Institute, Sant'Anna School of Advanced Studies, 56025 Pisa, Italy; ^3^Department of Excellence in Robotics & A.I., Sant'Anna School of Advanced Studies, Pisa 56127, Italy; ^4^Department of Agriculture, Food, and Environment, University of Pisa, 56124 Pisa, Italy; ^5^Healthcare Engineering Innovation Center (HEIC), Khalifa University, Abu Dhabi, UAE

## Abstract

Ontogenetic locomotion research focuses on the evolution of locomotion behavior in different developmental stages of a species. Unlike vertebrates, ontogenetic locomotion in invertebrates is poorly investigated. Locusts represent an outstanding biological model to study this issue. They are hemimetabolous insects and have similar aspects and behaviors in different instars. This research is aimed at studying the jumping performance of *Locusta migratoria* over different developmental instars. Jumps of third instar, fourth instar, and adult *L*. *migratoria* were recorded through a high-speed camera. Data were analyzed to develop a simplified biomechanical model of the insect: the elastic joint of locust hind legs was simplified as a torsional spring located at the femur-tibiae joint as a semilunar process and based on an energetic approach involving both locomotion and geometrical data. A simplified mathematical model evaluated the performances of each tested jump. Results showed that longer hind leg length, higher elastic parameter, and longer takeoff time synergistically contribute to a greater velocity and energy storing/releasing in adult locusts, if compared to young instars; at the same time, they compensate possible decreases of the acceleration due to the mass increase. This finding also gives insights for advanced bioinspired jumping robot design.

## 1. Introduction

Humans develop locomotion ability at the age of around one year [1]. On the contrary, a wide number of animals get their locomotion ability after being born [2]. This fact can be related to the prolonged parental care performed by humans compared to other animal species, in which juvenile individuals often face the same survival pressure as adult ones. In addition, it is well known that those juveniles are exposed to higher rates of mortality because of their smaller sizes and because of the hostile environment [3–6].

Ontogenetic locomotion research aims at answering two questions: (i) How do locomotion performances vary over different developmental stages? (ii) How may particular components of the locomotion system change during growth?

Allometric changes such as longer limbs, greater muscular force, greater contractile velocities, and muscular mechanical advantages can be easily observed in most juvenile vertebrates, improving their locomotion and boosting survival rates [2, 7–14]. However, although there is a wide number of researches focused on the ontogenetic locomotion ability in vertebrate animals [1, 7, 15–23], only a few studies are focused on invertebrate animals [2, 24–31], and most of them are focused on the jumping ability of locusts.

The jumping performance of different developmental instars in various locust species have been largely investigated [2, 27–29]. The allometric growth of the metathoracic leg, the increase in the mass of the femoral muscle relative to body mass, and the lengthening of the semilunar processes contribute to the augmentation of jumping performance from nymphs to adults [28]. The lengthening and thickening of the semilunar processes and the relative increase in the cross-sectional area of the extensor apodeme [27] work together to make up a stiffer spring system in the adult hind leg. In *Schistocerca gregaria* Forskål, this helps the hind legs of adults to store twofold energy, developing a higher takeoff velocity, i.e., >2.5 m/s, which is necessary to initiate flight in adults [27, 30].

The development and deposition of resilin in the energy storage component for locust jumping has been investigated by Burrows [32]. The thickness of the semilunar process and extensor resilin of newly molted instars and adults is initially thinner, then it increases because of resilin deposition after each molting, showing a general growing trend ontogenetically, while prior to a molt, the extensor resilin shows a declining trend. The jumping ability and performance of locusts at different life stages are consistent with the changes that occur during each molting cycle, which affect the energy store [32]. The energy stored during the deformation of the semilunar process, composites of hard cuticle and the rubber-like protein resilin, is around 50% of the jumping energy needed. In addition, it has been demonstrated that layered resilin/cuticle composites all share a similar distribution in the five nymphal stages and in adults in locusts [33]. This structure may be ubiquitous in jumping insects and play an important role in energy storing for jumping, in addition to the energy stored in the muscles.

The adults of the American locust, *Schistocerca americana* Drury, develop high-power, low-endurance jumps, while the juveniles perform less-power, high-endurance jumps [34], which is different from vertebrates [7, 15, 22, 35]. This can be explained by the fact that juvenile locusts use repeated jumping acts to escape from a wide number of their predators, with special reference to invertebrate ones (e.g., spiders and mantis) [29]. Besides, adults have to achieve a powerful jump to initiate flight in order to escape from faster predators, such as frogs, lizards, and birds, moving away with powerful flapping and gliding [29, 36–38]. The trade-off between jumping power and endurance is consistent with the ontogeny of life-history behaviors. However, juvenile locusts also use hopping as a model of locomotion exhibiting a difference between predator escape jumps and normal locomotion jumps [29, 34]. In this framework, the effects of the various instars on jumping performances of the African desert locust *S. gregaria* were investigated with an ontogenetic growth model [29]. Results show that force, acceleration, takeoff velocity, and kinetic energy, except power output, varied as an exponential function of body mass. Furthermore, a study on the effect of body mass and temperature on the jumping performance of *L. migratoria* indicates that jump energy scaled with body mass with a mean exponent of 1.15 across ontogeny and was otherwise unaffected by ambient temperature in the range of 15-35°C [39]. The energy stored by *L. migratoria* adults increases disproportionately from fifth instars and is greater over characterizing jumps of young instars, supporting results achieved on *S. gregaria* [29].

A few researches focused on ontogenetic locomotion development in invertebrates, and they specifically investigated the ontogenetic jumping performance of locusts [28, 29, 32, 34, 39–45]. However, little has been reported about the configurations of hind legs during the takeoff phase in locusts of different instars and their potential effect on the jumping performances. Based on this, the present study aimed to investigate if and how different hind leg configurations during the takeoff can affect the jumping performances in various developmental instars (i.e., third instar, fourth instar and adult). The geometrical parameters of *L. migratoria* individuals were combined with experimental data to set up a simplified mathematical model to assess the jumping performance of the tested locusts, and to explain the energy shift from *L. migratoria* nymphs to adults [2, 7, 27].

## 2. Materials and Methods

### 2.1. Experimental Setup and Material Preparation

A set of 29 *L. migratoria* adults, 17 fourth instars, and 35 third instars, was reared in different cylindrical transparent plastic boxes (50 cm in diameter and 70 cm in length) with a 16 : 8 (L : D) h photoperiod at 25 ± 1°C, 40 ± 5% RH. Temperature and RH conditions were the same during experiments. The health of each locust was constantly checked during the whole period assuring proper diet composed of wheat, fresh vegetables, and water *ad libitum* [44, 46, 47]. The experiments were carried out by using healthy locusts with no injuries (e.g., no damaged legs, wings, or antennae). The tested locusts were used at least 24 h after molting, to reduce the potential influence of soft newborn cuticles and small muscle mass on their locomotion and jumping performance [32, 39, 48].

All the locusts were weighed to 0.01 g with a scale. The dimensions of the main features (i.e., femur, tibiae, and tarsus length) were measured to the nearest 0.01 mm using a caliper.  Table 1 reports the results as mean value ± SD before testing their jumping performances.

A white-colored solid jumping platform was positioned inside a foam box (70 × 35 × 30 cm). The jumping platform was lit with four LED illuminators (RODER SRL, Oglianico TO, Italy) which emit red light (420 lm each at *k* = 628 nm) to match the maximum absorption frequency of the camera [49–53]. The jumping behavior of each locust was stimulated by teasing the rear of its body with a transparent plastic bar (2 mm diameter), to elicit the maximum “escape jumps.”

The jumps of each locust were recorded for 5 times interspersed by 10 minutes to allow the locusts to have a total recovery between jumps [43, 44]. Tested locusts jump from a prepared platform, and the body of locust body axis during the jumps is theoretically perpendicular to the axis of the camera. Jumps deviating more than 15° with respect to the perpendicular plane to the axis of the camera lens were excluded to limit the difference between the actual and perceived takeoff angle [39]. A Hotshot 512 sc high-speed camera (NAC Image Technology, Simi Valley, CA, USA) was used to record 2000 fps videos of the jumping tasks and store sequential 7600 images with a resolution of 512 × 512 pixels directly into its internal memory. All the samples were analyzed via the ProAnalyst Suite (Xcitex, Cambridge, MA, USA) to track the locust centroid trajectory for each jump.

### 2.2. Model Description

**Figure 1:**
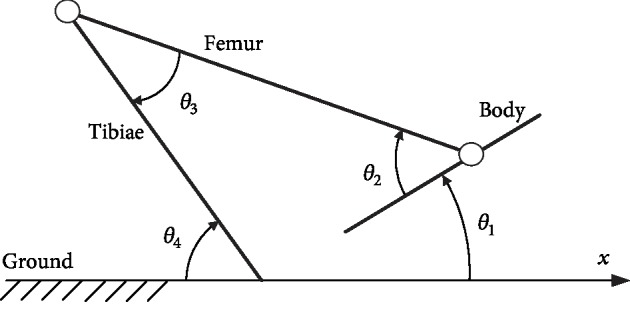
Simplified mechanical model of a *L. migratoria*.

A simplified mechanical model of a *L. migratoria* locust is depicted in Figure 1. The body, the femur, and the tibiae are outlined as three rigid bars. The *x* axis coincides with the ground, and *θ*_1_ is the angle between the body and the *x* axis: when the distance of the body line to the *x* axis increases positively, the value of *θ*_1_ is positive; otherwise, the value is negative. *θ*_2_ is the angle between the femur and body: when the femur line is upon the body line, the value of *θ*_2_ is positive; otherwise, the value is negative. *θ*_3_ is the angle between the femur and the tibiae. *θ*_4_ is the angle between the tibiae and the *x* axis. Both the values of *θ*_3_ and *θ*_4_ are strictly positive due to the structure of locust hind legs.

The cocking time was defined as the time interval needed for a *L. migratoria* individual to prepare to jump, from contracting the hind legs backward (*T*_1_) to being ready to jump (*T*_2_). The takeoff time is the time interval from the first observed movement of the hind leg (*T*_2_) to the detection of hind legs losing contact with the ground (*T*_3_). The release time is the time interval from the moment in which the hind legs lose contact with ground (*T*_3_) to the moment when the hind legs stop moving and are kept in a fixed position relative to body (*T*_4_).

Images of the jumping at *T*_1_, *T*_2_, *T*_3_, and *T*_4_, respectively, were carefully picked out from sequential images to evaluate the geometrical and temporal parameters (i.e., *θ*_1_, *θ*_2_, *θ*_3_, *θ*_4_, cocking time, takeoff time, and release time) via Microsoft Office Visio. The centroid of each locust was tracked during each jump by considering it positioned between the base of the middle and hind legs and bilaterally symmetrical from the vertical view [54].

The tracking paths were carefully checked to ensure that the tracking path corresponded to the raw image sequences [49]. Tracked center pixels of each video were converted into distances measured in millimeters with a scale ratio based on the graph paper and imported into the MATLAB software (MATLAB and Statistics Toolbox Release 2012b, The MathWorks, Inc., Natick, Massachusetts, United States).

### 2.3. Statistical Analysis

The influence of life stage on the considered parameters, i.e., the time intervals of different phases (cocking time, takeoff time, and release time), takeoff angle, legs' configuration over time, velocities at *T*_3_, *T*_4_, and *K* values (elastic parameters of tested jumps), and dimension parameters were analyzed separately using a general linear model with the following structure: *y* = *βX* + *ε*, where *y* is the vector of the observations with normal distribution (i.e., takeoff time, velocity at *T*_3_, or takeoff angle), *β* is the incidence matrix linking the observations to fixed effects, *X* is the vector of fixed effects (i.e., locust developmental instars), and *ε* is the vector of the random residual effects. ANCOVA (analysis of covariance) was used to analyze the effect of life stage on the jumping performance while considering body weight as a covariate, due to the fact that the difference of body weight is inevitable and the effect of body weight on the jumping performance should be elicited from the effect of life stage. A threshold *P* value of 0.05 was set to test the significance of differences between means. Post-hoc letters obtained by Tukey's HSD test separated averages.

## 3. Results

A set of 81 jump videos of different locusts (29 adults, 17 fourth instars, and 35 third instars) was analyzed with the abovementioned methods. The results for all the parameters are illustrated within the following subsections.

### 3.1. Time Intervals and Takeoff Angles of Tested Locusts

**Figure 2:**
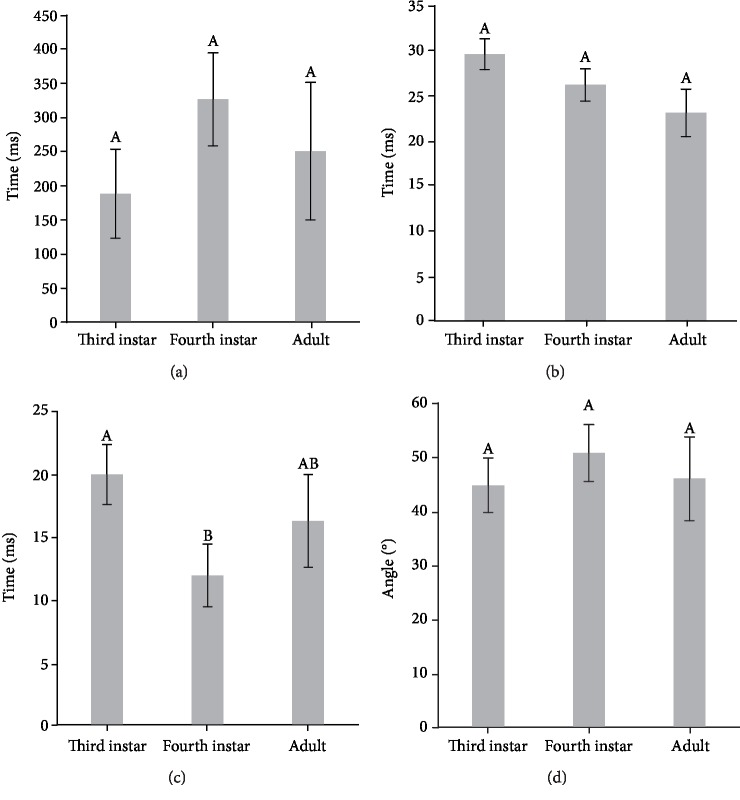
Mean cocking time (a), takeoff time (b), release time (c), and takeoff angles (d) of third instar, fourth instar, and adult *L. migratoria*. Different letters above each column indicate significant differences (*P* < 0.05). Whiskers represent standard errors.

The cocking time (*F*_2,80_ = 2.4780; *P* = 0.0906, Figure 2(a)) and takeoff time (*F*_2,80_ = 2.7304; *P* = 0.0715, Figure 2(b)) characterizing third instars, fourth instars, and adults of *L. migratoria* locusts showed no significant differences, while the release time ( *F*_2,80_ = 6.2732; *P* < 0.05, Figure 2(c)) showed significant differences among third instar, fourth instar, and adult locusts. The release time of third instar locusts was significantly longer than adult and fourth instar locusts (Figure 2(c)). The trajectory of the body center during the takeoff phase of *L. migratoria* was close to a straight line, and takeoff angle was defined as the slope angle of the body center trajectory of the tested locusts during takeoff [54, 55]. Considering the takeoff angle, no significant differences were detected in third instar, fourth instar, and adult locusts (*F*_2,80_ = 0.8065; *P* = 0.4502) .

*θ*_1_ and *θ*_2_ were significantly affected by the insect instars at *T*_2_. Both *θ*_1_ (*F*_2,80_ = 3.1813; *P* < 0.05, [Supplementary-material supplementary-material-1], in supplementary materials attached) and *θ*_2_ (*F*_2,80_ = 3.5052; *P* < 0.05) of adult locusts were significantly smaller than fourth instar locusts. *θ*_3_ (*F*_2,80_ = 7.5272; *P* < 0.05, [Supplementary-material supplementary-material-1]) of fourth instar locusts at *T*_3_ was significantly higher than that of third instar locusts. *θ*_3_ (*F*_2,80_ = 3.8030; *P* < 0.05) of third instar locusts at *T*_4_ was significantly smaller than fourth instar ones. *θ*_2_ (*F*_2,80_ = 5.8588; *P* < 0.05) and *θ*_4_ (*F*_2,80_ = 4.6958; *P* < 0.05, [Supplementary-material supplementary-material-1]) of fourth instar locusts were significantly smaller than third instar ones at *T*_4_. Based on the established simplified model in Figure 1, mean configurations of tested adult fourth instar and third instar locusts at *T*_1_, *T*_2_, *T*_3_, and *T*_4_ are plotted (Figures [Supplementary-material supplementary-material-1] in supplementary materials) using the mean dimension parameters (Table 1), center position tracking results, and mean angle data ([Supplementary-material supplementary-material-1]).

### 3.2. Velocities at *T*_3_ and *T*_4_ and Elastic Element Parameter of Tested Locusts

**Figure 3:**
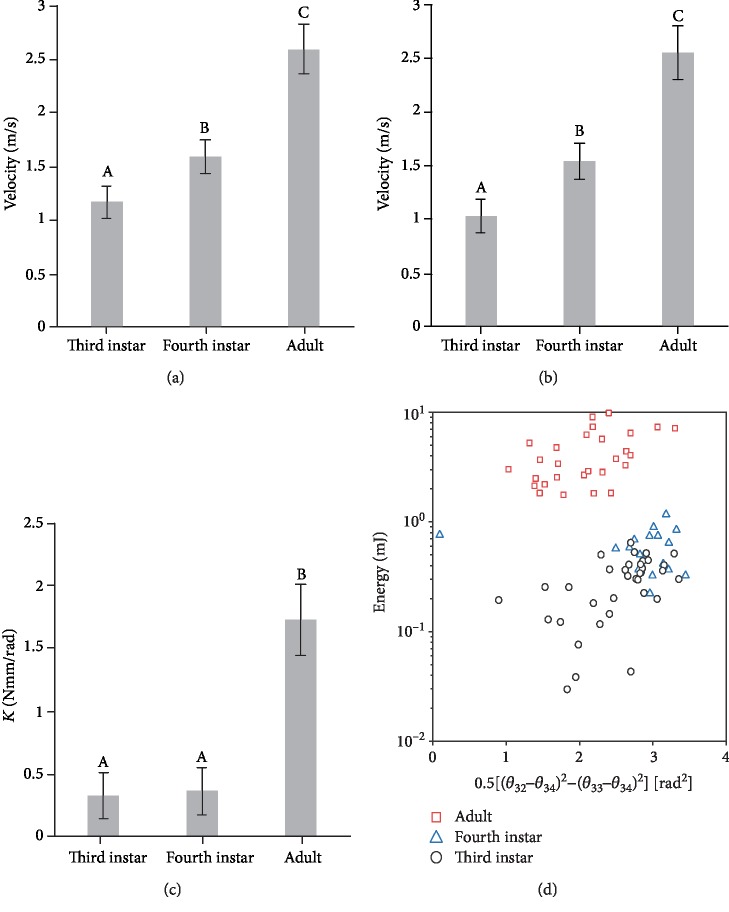
Mean velocities of third instar, fourth instar, and adult *L. migratoria* at *T*_3_ (a) and *T*_4_ (b). (c) Mean *K* value calculated based on Equation (2) of all tested third instar, fourth instar, and adult locusts separately. Different letters above each bar indicate significant differences (*P* < 0.05). Whiskers represent standard errors. (d) The relationship between the jump energy of locusts after takeoff phase at *T*_3_ moment and 0.5[(*θ*_32_ − *θ*_34_)^2^ − (*θ*_33_ − *θ*_34_)^2^]  of all tested third instar, fourth instar, and adult locusts' jumps.

The velocity at *T*_3_ (*F*_2,80_ = 9.8738; *P* < 0.05) and *T*_4_ (*F*_2,80_ = 10.5871; *P* < 0.0001) were significantly affected by the tested *L. migratoria* instar. The velocity at  *T*_3_ and  *T*_4_ of third instar individuals was significantly smaller when compared to that of adults and fourth instars (Figures 3(a) and 3(b)). For the third instar, fourth instar, and adult locusts, the mean velocities at *T*_3_ were bigger than the mean velocities at *T*_4_. The velocity decrease percentage from *T*_3_ to *T*_4_ of third instars (7.7%) is significantly higher (*F*_2,80_ = 4.3591; *P* < 0.05) and more than twice those of adults (3.3%) and fourth instars (2.19%) (Table 2).

The elasticity of the hind legs is simplified as a torsional spring (torsional stiffness: *K*) at femur-tibiae joints [56], the displacement in the vertical direction at *T*_3_ and *T*_4_ are *h*_3_ and *h*_4_, the mass of locust is *m*, and the gravitational acceleration is *g*, equals to 9.81 m/s^2^. The velocity of the mass center at *T*_2_ was set as 0 m/s, and the velocity of the mass center is *v*_3_ and *v*_4_ at *T*_3_ and *T*_4_, respectively. The values of *θ*_3_ at *T*_2_, *T*_3_, and *T*_4_ are *θ*_32_, *θ*_33_, and *θ*_34_, respectively. *θ*_34_ was considered to be the free position of the torsional spring. Based on energy conservation, the following formulas were used:

(1) $$\begin{array}{c} E_{3}=mgh_{3}+0.5mv_{3}^{2}, \end{array}$$

(2) $$\begin{array}{c} mgh_{3}+\frac{1}{2}mv_{3}^{2}=0.5K\left[{\left(\theta_{32}-\theta_{34}\right)}^{2}-{\left(\theta_{33}-\theta_{34}\right)}^{2}\right]. \end{array}$$

The energy of locusts at *T*_3_ and *T*_4_ were defined in Equation (1) individually, and the corresponding values were listed in Table 2. Based on Equation (2), elastic parameter *K* values of all tested jumps were calculated with known kinematic data and angle data. Elastic parameter *K* was significantly affected by the insect instars (*F*_2,80_ = 5.2980; *P* < 0.05), and *K* of tested adult locust jumps was significantly higher than that of fourth instar and third instar locust jumps (Figures 3(c) and 3(d)).

### 3.3. Hind Leg Length of Tested Locusts

The tibiae length of hind legs was significantly affected by *L. migratoria* instar (*F*_2,80_ = 24.4218; *P* < 0.001). The tibiae length of adults was significantly longer than that of fourth and third instars ([Supplementary-material supplementary-material-1], in supplementary materials attached). The femur length of hind legs was significantly affected by *L. migratoria* instar (*F*_2,80_ = 18.3199; *P* < 0.001). This femur length of adults was significantly longer than that of fourth and third instars ([Supplementary-material supplementary-material-1], in supplementary materials attached). The ratio of tibiae length to femur length of hind legs was not significantly affected by *L. migratoria* instars (*F*_2,80_ = 0.1378; *P* = 0.8715). The relation between mass and hind leg femur length and tibiae length of tested third instar, fourth instar, and adult locusts were included in the regression *L*_femur_ = 16.7880*m*^0.3144±0.0259^(*R*^2^ = 0.8806) and *L*_tibiae_ = 15.5597*m*^0.3299±0.0241^(*R*^2^ = 0.9033) individually ([Supplementary-material supplementary-material-1] and [Supplementary-material supplementary-material-1], in supplementary materials attached).

## 4. Discussion

How locust morphology can vary to fit the mutable mechanical demands of increasing body size and mass has been investigated by specific scaling models or allometries [27–29]. In our study, jumping performance related to some specific parameters (viz. tibiae length, body weight, and main joint angles) of *L. migratoria* individuals at different life stages were analyzed. Results showed that the jumping performance of *L. migratoria* adults outperformed those of young instars, both in terms of absolute velocity (Figure 3(a)) and mass specific work (Figure 3(f)).

**Figure 4:**
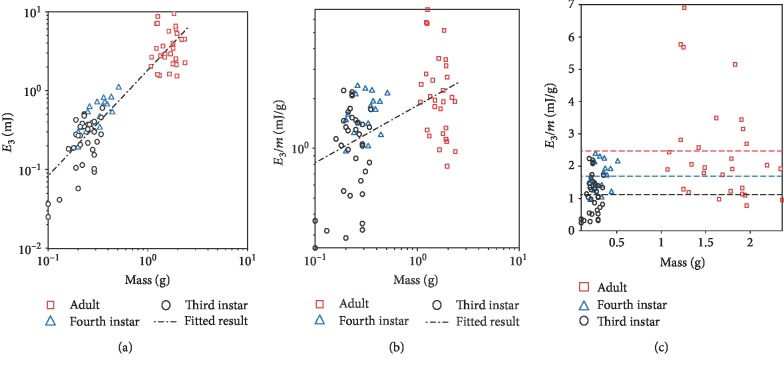
(a) The allometric relationship between jump energy and body mass of all tested third instar, fourth instar, and adult locust jumps. All tested jumps were included in the regression: *E*_3_ = 1.8018*m*^1.342±0.16^ (*R*^2^ = 0.8288). (b) The allometric relationship between mass-specific work (the ratio of jump energy divided by body mass) and body mass of all tested third instar, fourth instar, and adult locusts' jumps. All tested jumps were included in the regression: *E*_3_ = 1.8018*m*^0.342±0.136^  (*R*^2^ = 0.2388). (c) The allometric relationship between mass-specific work and body mass of all tested third instar, fourth instar, and adult locust jumps. The straight red, blue, and black lines are the average ratio of jumping energy divided by body mass of all tested third instar, fourth instar, and adult locusts individually.

Suppose *L. migratoria* locusts at different developmental instars follow a geometrically similar jump model [57], where both skeletal and muscular properties obey the laws of geometric scaling—“muscle work”—which means the energy delivered during the push-off should scale at the same rate of mass and mass-specific works are independent of scale. Two conclusions should be obtained based on this model: (i) The specific energy (*E*_3_/*m*) should be the same for all tested instars and adults of *L. migratoria*. (ii) Due to a size effect, small size jumpers, such as fourth and third instar locusts, should reach similar (or slightly higher) takeoff velocities if compared to adult locusts. However, experimental results disagree with both conclusions. Firstly, mass-specific works showed an increasing trend during growing (Figures 4(b) and 4(c)). Secondly, even though adult locusts have bigger masses than younger instar ones (Table 2), adults have significantly larger takeoff velocities (Figure 3(a)). These apparent paradoxes showed that *L. migratoria* locusts at different developmental instars cannot be expected to perform as geometrically similar jumpers.

The relation between mass and energy of all tested jumps of third instar, fourth instar, and adult locusts (Figure 4(a)) was included in the regression: *E*_3_ = 1.8018*m*^1.342±0.16^ (*R*^2^ = 0.8288). Similar regression values were concluded in two separate studies: *E*_3_ = 1.91*m*^1.14±0.09^ (*R*^2^ = 0.96) for *L. migratoria* juveniles [39] and  *E*_3_ = 1.7906*m*^1.114^ (*R*^2^ = 0.939) [29] for *S. gregaria* juveniles. In both published research, adult locusts' jumps have greater kinetic energy than the value predicted using the regression that concluded using only juveniles; for example, *S. gregaria* adults produces around four times as much kinetic energy as the regression predicted for juveniles using adult body mass [29]. In our experiment, the regression included tested jumps of both adult and instar locusts, because the regression using only juveniles is *E*_3_ = 3.5278*m*^1.8018±0.4840^ (*R*^2^ = 0.5473) and the coefficient of determination *R*^2^ is 0.5473, which is relatively lower than that including both adult and instar locusts (*R*^2^ = 0.939). If we adopt the regression using only juveniles to predict the kinetic jumping energy of adults, the predicted jumping energy is greater than the real kinetic energy, which is different from previous published results.

Considering the state of the art, the authors examined the scaling of jumping performance in *L. migratoria* to understand whether there is a connection between functional and morphological designs. Even though adult locusts have body masses bigger than fourth instar and third instar locusts (Table 2), adults have significantly higher velocity and energy after takeoff (Table 2). This result is comparable with existing research focused on *S. gregaria.* Juvenile *S. gregaria* locusts produce takeoff velocities of 0.9-1.2 m/s, while adult locusts show takeoff velocities around twice as high as that of juveniles (2.5 m/s) [29]. In addition, the kinetic energy of the jump in *S. gregaria* have values that range from a low of 0.004 mJ in a first-instar locust to as high as 15.99 mJ in an adult [29]. The greater takeoff velocity in adult locusts and excellent jumping performance can be explained by different reasons.

Firstly, the lengths of the femur and tibiae show a significant increase in *L. migratoria* individuals during its development, in a comparable manner to results previously achieved on *S. gregaria* [42]. Jump distance is demonstrated to be proportional to the distance through which the force acts [41, 58, 59], which is related directly to limb length. Thus, the relatively longer legs (including the femur and tibiae) of older juveniles likely provide the approaches to propel these animals farther and with greater jump energy [39]. In addition, the muscle mass in the femurs of adult locusts shows a higher percentage of body mass compared to those in young instar locust [27] and shows an aligned increase in the angle of muscle pennation [28]; both lead to a greater capacity for energy storage and greater jump velocity [28].

The importance of tibiae mechanical property has been investigated [29, 42], and the authors pointed out that the increase of tibiae length in *S. gregaria* during growth can help locusts to adapt to the acceleration decrease caused by the increase of body mass [42] with an enlarged takeoff time in adults. James et al. also reported that the increased relative hind limb length and relative mass of jumping muscles ensure the improvement of jumping performance [58]. In contrast, Katz and Gosline stressed that tibiae play an important role during the takeoff phase and work like a bending spring rather than a rigid bending lever in *S. gregaria* [42]. The obvious deflection of tibiae during takeoff [60] can store at least 10% of the total kinetic energy of the jump [42]. The effect of leg compliance on jumping performance is also investigated in jumping robots [61, 62], and results demonstrated that proper leg compliance can improve the performance of a jumping robot using the initially stored energy in the compliant legs to be used. Based on this, the significant increase in tibiae length were considered meaningful to improve jumping performances of adult locusts.

Secondly, the established mechanical model revealed that locust adults have a significantly bigger *K* value if compared to fourth instar and third instar individuals (Figure 3(c)). This seems directly connected with the better jumping performance characterizing adult locusts. A sharp improvement of velocity and energy in adults is reported to be a result of the combination of a bigger mean cross-sectional area of the femur muscle [28, 63, 64] coupled with the fact that a rather long life span gives adult locusts longer time to stiffen their semilunar process and extensor cuticle [27, 32, 40, 65, 66]. A stiffer spring system in adults was also estimated by the abovementioned modeling, where the elasticity of the hind legs of locusts was supposed to be modeled as a torsional spring located at the femur-tibiae joint [56], neglecting other elastic contributions [67, 68]. The results showed that the stiffness of fourth instar and third instar locusts are close, and rather smaller than that of adults, differing by orders of magnitude.

Finally, due to the viscoelasticity of muscular tissues, longer takeoff times in adults *L. migratoria* decrease the energy consumption during takeoff caused by internal dissipative forces.

**Figure 5:**
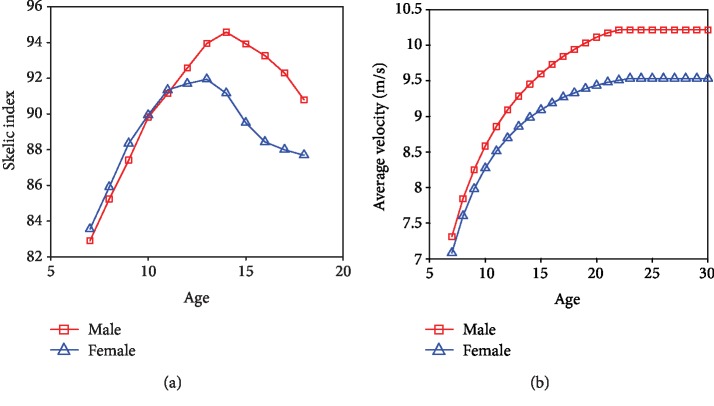
(a) The development curve of skelic index and age [69]. (b) The development curve of average velocity of 100 m sprint best record and age [70].

There is an interestingly similar phenomenon in locusts and humans. The value of 0.5[(*θ*_32_ − *θ*_34_)^2^ − (*θ*_33_ − *θ*_34_)^2^] in fourth instar locusts showed an increasing trend compared to third instar ones, while for adult locusts it showed a decreasing trend (Figure 3(d)). This is very similar to the skelic index (standing height minus sitting height divided by sitting height and multiplied by 100) development trend in human beings (Figure 5(a)); the skelic index has its maximum value at around 15 years old and then decreases. Both the takeoff velocity of tested locusts (Figure 3(a)) and average velocity of 100 m sprint best record (Figure 5(b)) showed an increasing trend until becoming adults. The best locomotion performance for both locusts and humans happens in adults. It likely conveys that the locomotion performance is a combined result of both geometrical parameters and material property. For adults, the best geometrical parameters (0.5[(*θ*_32_ − *θ*_34_)^2^ − (*θ*_33_ − *θ*_34_)^2^] for locusts and skelic index for humans) and best material property (muscle occupation rate and elastic parameters) are achieved simultaneously and result in the best locomotion performance.

Interestingly, the percentage velocity difference from *T*_3_ to *T*_4_ strongly increases as the body size decreases, from adults (3.3%) and fourth instar (2.19%) to third instar locusts (7.7%) (Table 2). This phenomenon may be connected to the fact that smaller instars have a higher frontal area-to-body mass ratio compared to larger instars, which makes them more susceptible to the effects of aerodynamic drag [39, 71]. Another possible reason is the longer release time in third instar locusts. The takeoff angles in all tested locusts are similar, close to 45°, helping to maximize the jumping distance [39, 72, 73].

In agreement with the findings obtained studying *S. americana* [34], the compromise between power and endurance was noticed in the present research. Indeed, *L. migratoria* adults took a longer time than 10 minutes to be ready for the next jump. In several instances, after recording a powerful jump in an adult locust, it was hard to record another one, while the situation was different for fourth instar and third instar locusts. After a less powerful jump, it took less time before the next one was ready for another jump and they were willing to jump another time if stimulated again within a short time interval.

Overall, we detected a longer takeoff time in adult locusts, if compared to young instars, although the velocity was higher and the release time shorter, probably to allow the spread of wings to start the flight. Locusts can learn motor actions at the level of the single ganglia [74]. Therefore, a longer takeoff time, as well as a higher velocity and a shorter release time, could be chiefly influenced by their increasing motor experience from young instar to adult. Furthermore, the specialization of leg control seems to be related to particular neural circuits involved in sensory-motor mechanisms occurring within the prothoracic ganglion of these insects [75, 76]. In addition, adults were found to be more efficient in storing energy in their hind legs and releasing it during the jump. Indeed, *K* of tested adults' jumps were significantly higher compared to that of fourth and third instars: this could be related to a more efficient composite storage device, consisting of a greater mass of soft resilin and a thicker hard cuticle in adult locomotor structures due to growth, contributing to adults with enhanced performances during the jumping behavior [77–79]. Further research is still needed to shed light on the abovementioned issue.

In conclusion, velocity after takeoff and energy per jump are significantly higher in adult locusts over the fourth and third instars, while the body mass of adult locusts is a half magnitude bigger than the fourth and third instar ones. This is compensated by peculiar morphological design and stiffness. Longer hind legs boost the acceleration time and compensate for the supposed acceleration decrease [29, 42]. A bigger tibiae-to-femur ratio means a relatively longer tibiae, supporting the prediction that the tibia works as a leaf spring and the deflection of tibiae can store a significant part of energy needed by each jump. The spring system of locust hind legs is composed by elastic cuticles and a semilunar process. The thickness of the semilunar process and extensor resilin show a general increasing trend during development, while decreasing during molting [32]. The stronger spring system in adult locusts is consistent with the calculation results based on a simplified mathematical model proposed here. The stiffer spring system and bigger muscle occupation rate work together to improve the adult locust jumping performance [32]. This study adds basic knowledge on the jumping mechanisms in various developmental instars of *L. migratoria* locusts considering a different leg configuration as well as body mass, length of hind legs, velocity, and energy. We also proposed a simplified mathematical model to calculate the elastic features of each jump in young instars and adults of *L. migratoria*.

The ontogenetic jumping performance of locusts reported here can inspire roboticists to select the most suitable instars as a model organism to design advanced jumping robots. Firstly, jumping represents the only locomotion mode (e.g., early instar locusts) or can be coupled with flapping and gliding wings (e.g., adult locusts). Secondly, the mass (Table 1), the consumed energy (Table 2), and the elastic parameter *K* (Figure 3(c)) increase around one order of magnitude from third instar to adult locusts, which convinces us that size and weight are key parameters in jumping robot design together with the elastic and actuation systems. Thirdly, it is important to consider geometrical parameters in robots' design, due to the significant variation of geometrical parameters (e.g., joint angles, tibiae length and the ratio of tibiae length to femur length of hind legs) in locusts and their impact on jumping performance.

## Figures and Tables

**Table 1:** Weight and linear dimension parameters characterizing the tested third instars, fourth instars and adults of *Locusta migratoria*.

Stage	Weight (g)	Body (mm)	Femur (mm)	Tibiae (mm)	Tarsus (mm)	Samples
Third instar	0.24 ± 0.06	19.83 ± 1.58	10.58 ± 1.12	9.58 ± 0.94	3.90 ± 0.46	35
Fourth instar	0.32 ± 0.09	23.56 ± 2.02	11.24 ± 0.93	10.21 ± 0.81	4.23 ± 0.54	17
Adult	1.65 ± 0.36	44.61 ± 3.71	20.06 ± 1.69	18.72 ± 1.51	6.79 ± 0.91	29

**Table 2:** Mean velocity and energy of tested *L. migratoria* at *T*_3_ and *T*_4_ moments.

Type	Weight (g)	*E*_3_ (mJ)	*E*_4_ (mJ)	*v*_3_ (m/s)	*v*_4_ (m/s)	(*v*_3_ − *v*_4_)/*v*_3_
Third instar	0.24 ± 0.06	0.27 ± 0.15	0.24 ± 0.15	1.43 ± 0.42	1.32 ± 0.43	7.7%
Fourth instar	0.32 ± 0.09	0.55 ± 0.24	0.55 ± 0.27	1.82 ± 0.25	1.78 ± 0.32	2.19%
Adult	1.65 ± 0.36	3.89 ± 2.21	3.71 ± 2.38	2.12 ± 0.65	2.05 ± 0.69	3.30%

## Data Availability

The excel data used to support the findings of this study are available from the corresponding author upon request.

## Supplementary Materials

Supplementary Materials Figure S1: (a) the mean values of *θ*_1_, *θ*_2_, *θ*_3_, and *θ*_4_ of tested adult, fourth instar, and third instar locusts separately at *T*_1_ (a), *T*_2_ (b), *T*_3_ (c), and *T*_4_ (d). Asterisks indicate significant differences. “^∗^” and “^∗∗^” coexisting means that those two items are significant to each other while no significance exists between those two items and another one. *T*-bars represent standard errors. Figure S2: mean configurations of tested (a) adult, (b) fourth instar, and (c) third instar locusts separately based on the mean dimension parameters (Table 1), center position tracking results, and mean angle data at *T*_1_, *T*_2_, *T*_3_, and *T*_4_ (Figure S1). The relative position of the body, femur, and tibiae are simplified based on the model established in Figure 1. The mean configurations of tested locusts at *T*_1_, *T*_2_, *T*_3_, and *T*_4_ are drawn in black, red, blue, and pink lines separately. Figure S3: (a) the mean values of hind leg tibiae length of tested adult, fourth instar, and third instar locusts separately; (b) the mean values of hind leg femur length of tested adult, fourth instar, and third instar locusts separately. Different letters above each column indicate significant differences (*P* < 0.05). Whiskers represent standard errors. Figure S4: (a) the allometric relationship between hind leg femur length and body mass of all tested third instar, fourth instar, and adult locust jumps. All tested locusts were included in the regression: *L*_femur_ = 16.7880*m*^0.3144±0.0259^ (*R*^2^ = 0.8806); (b) the allometric relationship between hind leg tibiae length and body mass of all tested third instar, fourth instar, and adult locust jumps. All tested locusts were included in the regression: *L*^tibiae^ = 15.5597*m*^0.3299±0.0241^ (*R*_2_ = 0.9033).
