# Supplementary material for: Impact of Different Developmental Instars on Locusta migratoria Jumping Performance
Source: Appl Bionics Biomech. 2020 Mar 19;2020:2797486. doi: 10.1155/2020/2797486 (PMC7136764; doi:10.1155/2020/2797486)
Supplement: Supplementary Materials — Figure S1: (a) the mean values of θ1, θ2, θ3, and θ4 of tested adult, fourth instar, and third instar locusts separately at T1 (a), T2 (b), T3 (c), and T4 (d). Asterisks indicate significant differences. “∗” and “∗∗” coexisting means that those two items are significant to each other while no significance exists between those two items and another one. T-bars represent standard errors. Figure S2: mean configurations of tested (a) adult, (b) fourth instar, and (c) third instar locusts separately based on the mean dimension parameters (Table 1), center position tracking results, and mean angle data at T1, T2, T3, and T4 (Figure S1). The relative position of the body, femur, and tibiae are simplified based on the model established in Figure 1. The mean configurations of tested locusts at T1, T2, T3, and T4 are drawn in black, red, blue, and pink lines separately. Figure S3: (a) the mean values of hind leg tibiae length of tested adult, fourth instar, and third instar locusts separately; (b) the mean values of hind leg femur length of tested adult, fourth instar, and third instar locusts separately. Different letters above each column indicate significant differences (P < 0.05). Whiskers represent standard errors. Figure S4: (a) the allometric relationship between hind leg femur length and body mass of all tested third instar, fourth instar, and adult locust jumps. All tested locusts were included in the regression: Lfemur = 16.7880m0.3144±0.0259 (R2 = 0.8806); (b) the allometric relationship between hind leg tibiae length and body mass of all tested third instar, fourth instar, and adult locust jumps. All tested locusts were included in the regression: Ltibiae = 15.5597m0.3299±0.0241 (R2 = 0.9033). [file 2797486.f1.pdf]

Supplementary Materials

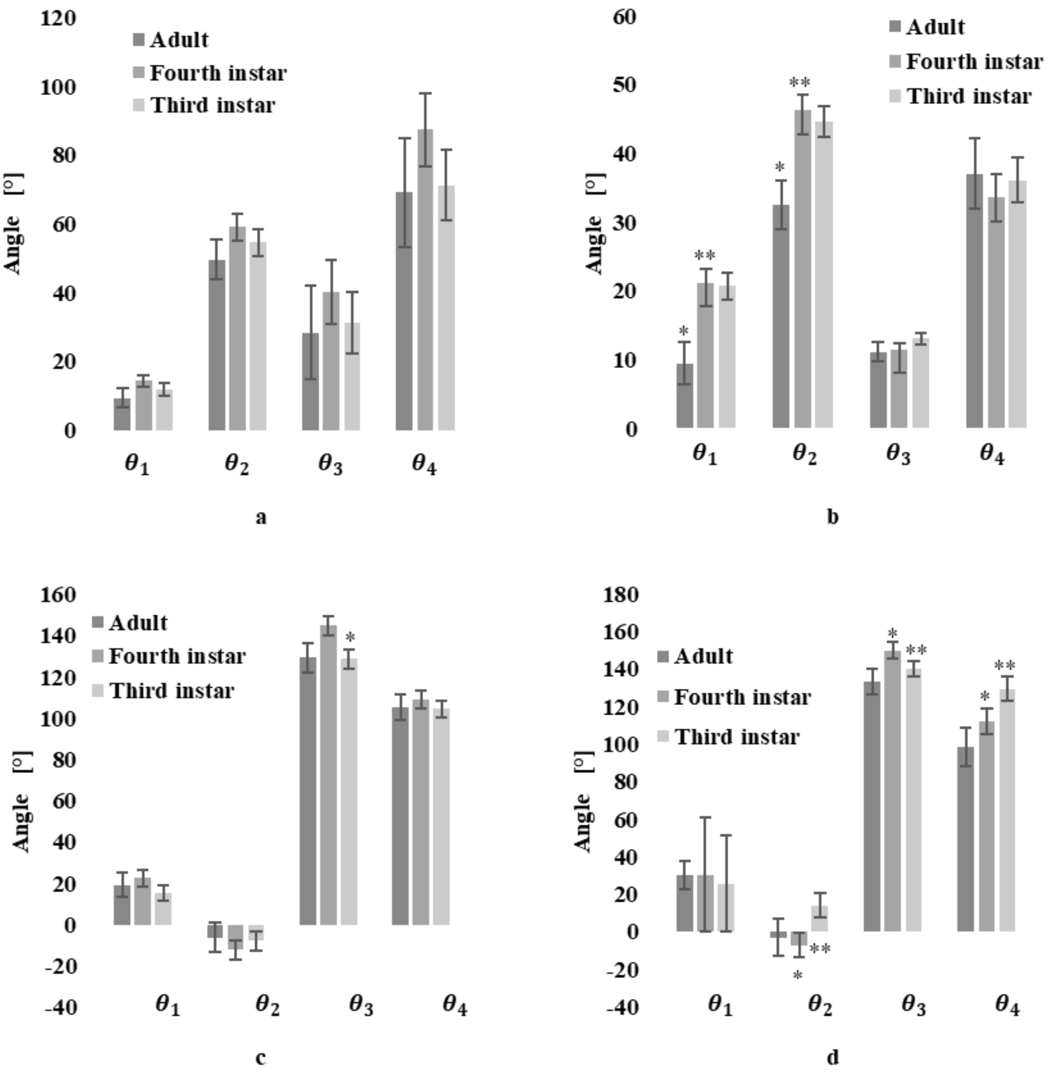

Figure S1. (a) The mean values of θ_1, θ_2, θ_3 and θ_4 of tested adult, fourth instar and third instar locusts separately at T_1 **(a)**, T_2 **(b)**, T_3 **(c)**, T_4 **(d)**. Asterisks indicate significant differences. '*' and '**' coexisting means that those two items are significant to each other while no significance exists between those two items and another one. T-bars represent standard errors.

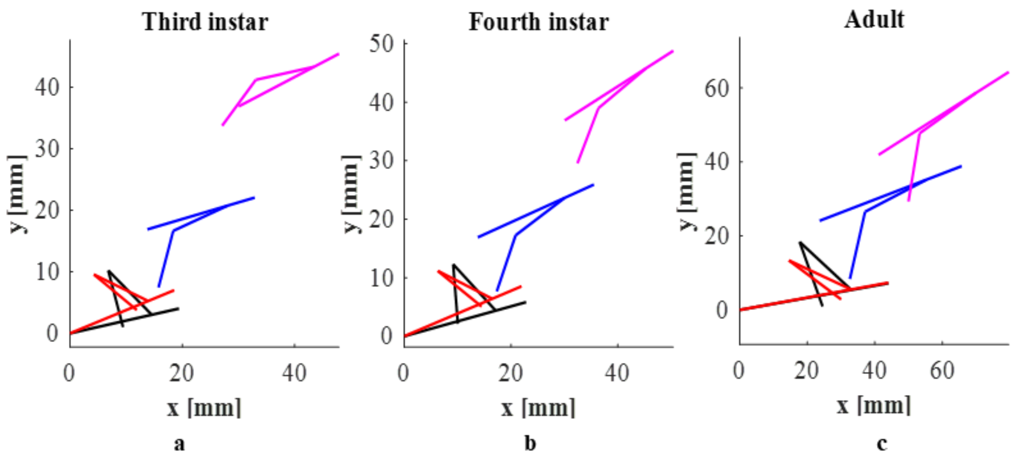

Figure S2. Mean configurations of tested (a) Adult (b) Fourth instar and (c) Third instar locusts separately based on the mean dimension parameters (Table 1), center position tracking results and mean angles data at T_1, T_2, T_3 and T_4 (Figure S1). The relative position of body, femur and tibiae are simplified based on the model established in Figure 1. The mean configurations of tested locusts at T_1, T_2, T_3 and T_4 are drawn in black, red, blue and pink lines separately.

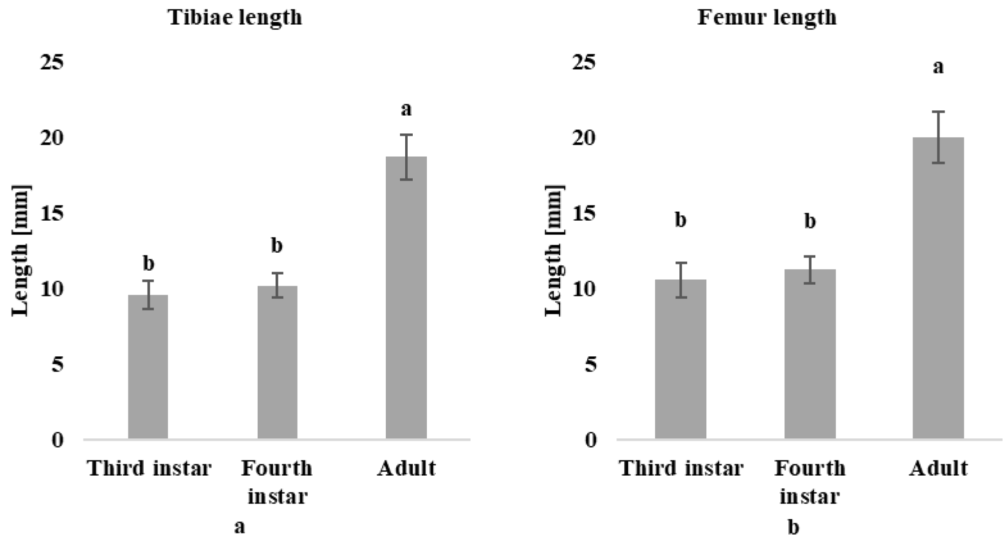

Figure S3. **(a)** The mean values of hindlegs tibiae length of tested adult, fourth instar and third instar locusts separately; **(b)** The mean values of hindlegs femur length of tested adult, fourth instar and third instar locusts separately. Different letters above each column indicate significant differences ($P<0.05$). Whiskers represent standard errors.

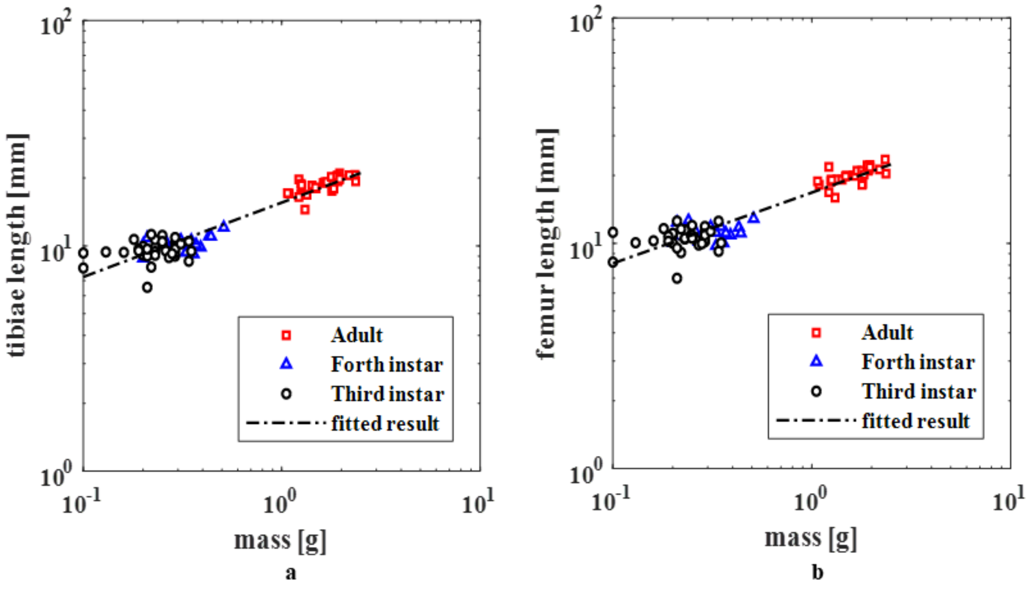

Figure S4. **(a)** The allometric relationship between hindleg femur length and body mass of all tested third instar, fourth instar and adult locust jumps. All tested locusts were included in the regression: $L_{femur} = 16.7880m^{0.3144\pm0.0259}(R^2 = 0.8806)$; **(b)** The allometric relationship between hindleg tibiae length and body mass of all tested third instar, fourth instar and adult locust jumps. All tested locusts were included in the regression: $L_{tibiae} = 15.5597m^{0.3299\pm0.0241}(R^2 = 0.9033)$.
